# Access to a Newly Adapted Diabetes-Specific Multimodal Cross-Sectoral and Interdisciplinary Psychotherapeutic Care Option for a High-Risk Group of Patients With Diabetes Mellitus and Distress: Protocol for the minDBe Pilot Study

**DOI:** 10.2196/73199

**Published:** 2025-10-17

**Authors:** Sandra Zara, Johannes Kruse, Hanna Kampling

**Affiliations:** 1 Department of Psychosomatic Medicine and Psychotherapy University of Giessen Giessen Germany

**Keywords:** glycemic control, psychological burden, mixed methods, patient perspective, service provider perspective, multimodal treatment

## Abstract

**Background:**

Diabetes is a chronic disease requiring daily self-management to regulate physiological parameters like glycemic control. Despite extensive training being available, most patients with diabetes do not meet their target blood sugar levels. Moreover, diabetes-related emotional distress and psychological burden in the form of depressive and anxiety symptoms are very common. Patients with these challenges represent a high-risk group in terms of increased morbidity, mortality, and health care costs as well as decreased quality of life. Hence, in addition to somatically focused standard care, this patient group requires specific treatment options that also address psychosocial aspects. However, these treatments are often not available in routine care. Psychosomatic outpatient clinics (PsIAs) could address this gap by offering multimodal and integrative treatment. However, the indication for treatment in PsIAs and the referral pathways for patients with diabetes from practices or diabetes clinics to PsIAs remain complicated. Moreover, specific treatment options must be adapted.

**Objective:**

This study aims to assess the needs for referral pathways to PsIAs from the patient and service provider perspectives and to adapt the already effective psychosomatic intervention psy-PAD for a group setting (psy-PAD_Group_), fitting the multimodal PsIA structures.

**Methods:**

This pilot study comprises an explorative qualitative design based on a multilevel approach, using 2 work packages: work package 1 assesses the patient perspective (n=40) using focus groups, as well as the service provider perspective with interviews (n=30). Work package 2 comprises an expert workshop with clinicians and patient representatives (n=10) to adapt the psy-PAD_Group_ manual for the modalities of PsIAs. We will audio-record the patient focus groups and service provider interviews. The audio recordings will be transcribed and analyzed using content analysis within the framework of interpretative text evaluations with MAXQDA.

**Results:**

Outcomes will be (1) the needs regarding referral pathways, (2) short questionnaires to economically assess the acceptance and feasibility of the referral pathways in preparation for the minDBe main study, and (3) the adapted psy-PAD_Group_ intervention for an interdisciplinary multimodal treatment option to be implemented in the structures of PsIAs.

**Conclusions:**

The minDBe pilot study will lay the foundation for the minDBe main study, which aims to evaluate the psy-PAD_Group_ as part of an interdisciplinary multimodal psychotherapeutic treatment program in a randomized controlled trial.

**International Registered Report Identifier (IRRID):**

DERR1-10.2196/73199

## Introduction

### Background

Diabetes mellitus is a highly prevalent, chronic, noncommunicable disease with a global prevalence of 6.2% and 10% in Germany [1,2]. Among other considerations, maintaining quality of life and preventing diabetes-associated secondary diseases is a central goal in diabetes treatment. To achieve this goal, daily diabetes self-management is required to keep glycemic control (hemoglobin A_1c_; HbA_1c_) close to or lower than 7.5% [3]. However, in Germany, around 40% of patients with type 1 diabetes and 58% of patients with type 2 diabetes do not achieve their target HbA_1c_ values [4], despite comprehensive diabetes training and structured disease management programs being available.

Factors that have been associated with poor glycemic control are psychological burden as well as diabetes-related distress [5]. A bidirectional relationship between diabetes and depressive and anxiety disorders is widely established [6-9]. Depressive disorders were significantly higher in patients with diabetes compared to those without (type 1: 22% vs 13% and type 2: 19% vs 11%) [10]. The risk of developing anxiety disorders and elevated anxiety symptoms is twice as high for patients with diabetes compared to individuals without diabetes [11]. Moreover, patients with diabetes often experience diabetes-related distress, defined as the general emotional distress of managing diabetes as well as specific diabetes-related worries [12]. While to a certain extent it overlaps with depressive symptoms, diabetes-related distress is a clearly delineated construct [13]. Both depressive and anxiety symptoms and diabetes-related distress are associated with problematic self-management, a lower quality of life, and poor glycemic control [5,13-17]. In addition, they are linked to an increased risk of secondary diseases and diabetes-associated complications [14,18], premature mortality [17,19], as well as significantly increased health care costs [20]. Hence, psychosocial aspects of diabetes are increasingly coming into treatment focus [21].

Given the close association between poor glycemic control, psychological burden, and diabetes-related distress, various studies tried to address this issue by applying psychological, psychotherapeutic, and psychopharmacological interventions to improve both psychological burden on the one hand and somatic outcomes such as glycemic control on the other hand. While some studies have indeed shown the effectiveness regarding psychological outcomes, there are heterogeneous results regarding their effectiveness for glycemic control [22-24]. These findings emphasize that treating psychological burden alone does not generally have a positive effect on glycemic control. Hence, general interventions not considering diabetes-specific challenges, such as diabetes-related distress, do not seem to be enough to improve glycemic control as well. Moreover, addressing psychological burden and somatic specifications (including diabetes-related distress) as part of an integrative treatment seems promising [25]. Internationally, integrative treatment approaches that allow for access to structured, interdisciplinary care concepts integrated into primary care have already proven to be effective for patients with diabetes in reducing psychological burden and also glycemic control [26].

In Germany, neither integrative treatment options with access to structured interdisciplinary care nor interventions addressing psychological burden and diabetes-related distress are usually available in standard routine care. Patients with diabetes are mainly treated by their general practitioner, by their diabetologists, or in specialized diabetes clinics, while individuals with psychological burden usually seek treatment in psychotherapeutic settings (inpatient clinics, day care clinics, or outpatient psychotherapist practices) [27]. This implies that most patients with diabetes receive either somatic or psychotherapeutic treatment, which are not aligned with each other (even if they are received simultaneously). Against the background of this fragmented health care landscape, which generally represents a challenge in the treatment of different patient groups, psychosomatic outpatient clinics (PsIAs) were newly implemented in 2019 in Germany. PsIAs are outpatient treatment options that do not require inpatient or day clinic stay but allow for a higher treatment intensity normally found in inpatient settings. They comprise complex treatment options integrating highly specialized multiprofessional treatment addressing both somatic and psychosocial aspects as well as disease-specific challenges. About the specific requirements of a certain patient group, for example, patients with diabetes, PsIAs can offer indication-specific services that are part of a multimodal and interdisciplinary treatment. A recent randomized controlled trial using an integrated psychosomatic treatment program for patients with diabetes (the psy-PAD intervention) could show effectiveness for this patient group regarding poor glycemic control as well as diabetes-related distress [25]. PsIAs offer the opportunity to implement such an intervention while also offering a more comprehensive multimodal, interdisciplinary, and integrated treatment setting.

### Challenges and Objectives

However, for such an implementation, numerous challenges arise. For one, specific adaptations of such a treatment option are necessary to fit the structures of PsIAs. Moreover, the referral pathways to PsIAs pose a challenge, as various specialists are involved. Patients with diabetes need to be identified to also experience psychological burden and diabetes-related distress (usually during standard somatic care by diabetologists), and moreover, they actually need to be diagnosed with a mental disorder by a specialist (usually as part of an additional appointment with a medical specialist for psychosomatic medicine, psychiatry, or psychotherapy). However, such referral pathways are not yet established, and more, they are especially difficult to follow due to the predominantly somatic understanding of their disease. Against this background, the minDBe pilot study has 2 aims. First, developing referral pathways to PsIAs and short questionnaires to economically assess the feasibility of these referral pathways, and second, adapting and further developing the already tested and manualized psy-PAD intervention [25] addressing patients with diabetes and poor glycemic control as well as diabetes-related distress for a group setting (psy-PAD_Group_).

## Methods

### Study Design

**Figure 1:**
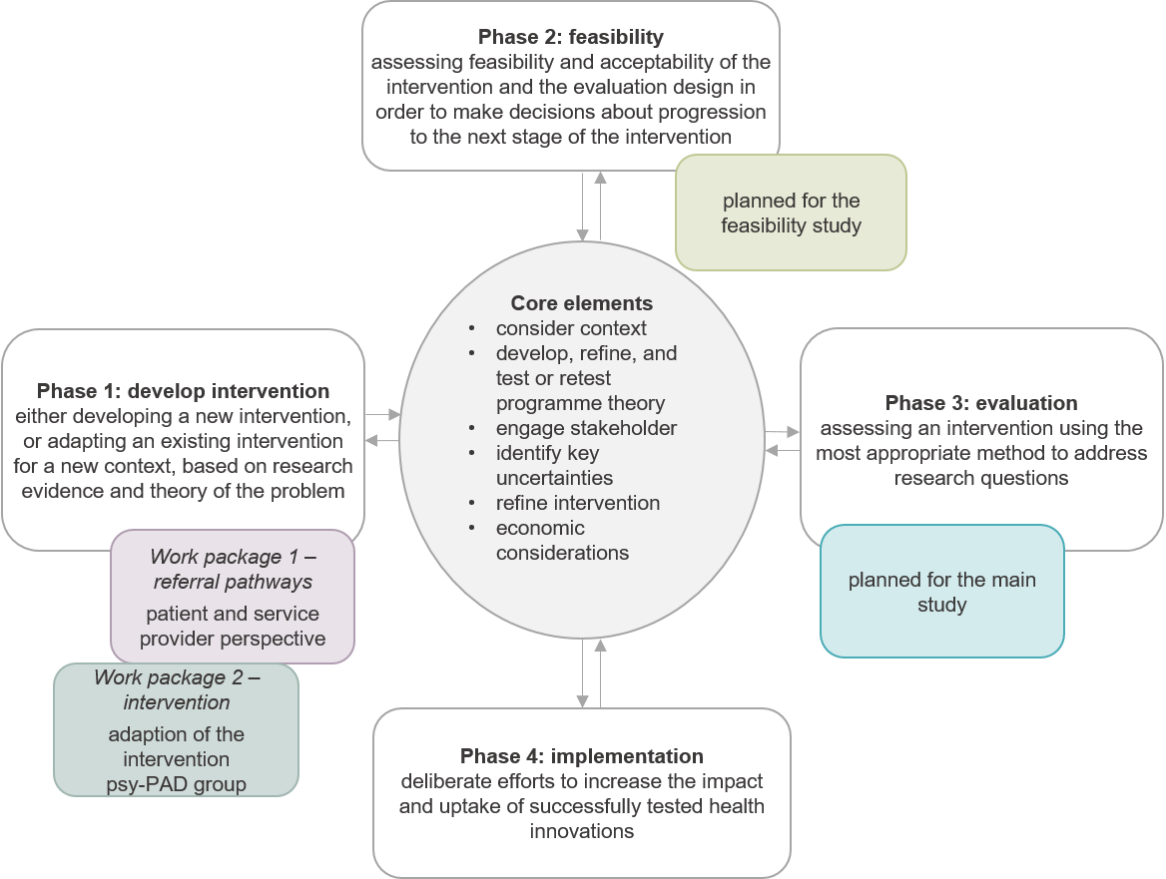
Inclusion of the work packages and the planned feasibility and main study in the medical research council framework. psy-PAD group: psy-PAD for a group setting.

**Figure 2:**
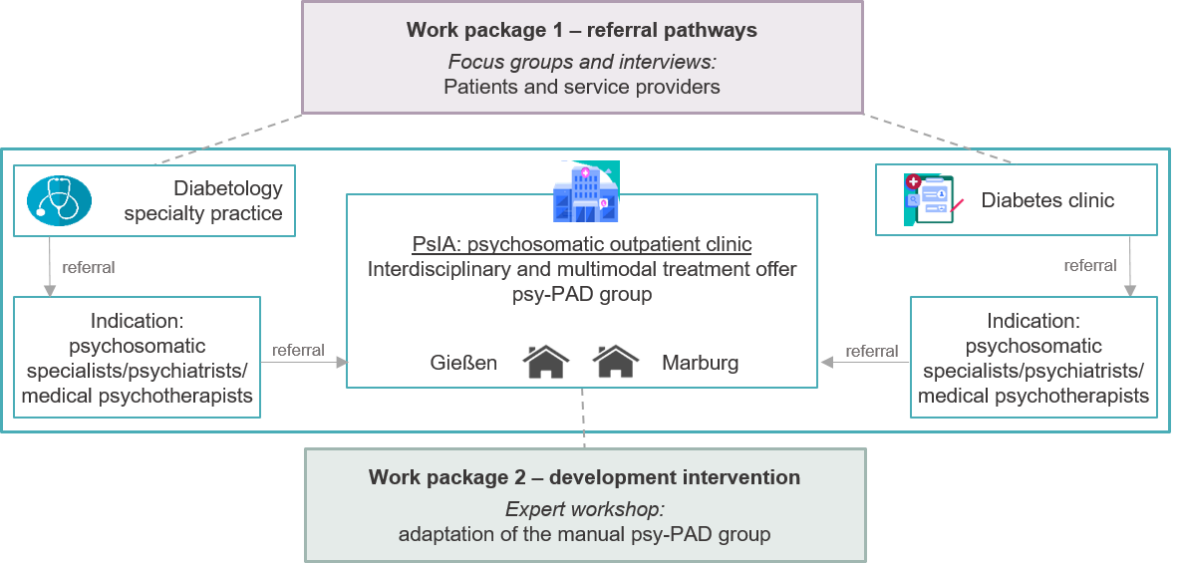
Study design—overview of the 2 work packages in the overall project and the allocation of the methods used. psy-PAD group: psy-PAD for a group setting.

The minDBe pilot study comprises a complex intervention by integrating different methodological approaches. Against the background of the medical research council framework published by the UK Medical Research Council [28], the minDBe pilot study encompasses an explorative qualitative research design based on a multilevel approach (patients and service providers) in 2 work packages (Figures 1 and 2). Work package 1 addresses the needs for referral pathways to PsIAs from both the patient and the service provider perspectives. It aims to develop short questionnaires to economically assess the feasibility of these referral pathways in preparation for the upcoming minDBe main study. The patients’ and service providers’ needs regarding the implementation of a diabetes-specific care option within PsIAs, as well as the referral to PsIAs, will be qualitatively examined with patient focus groups and service provider interviews, respectively. The focus will be on acceptance, feasibility, barriers, and facilitators.

Work package 2 aims to adapt the psy-PAD intervention into the group intervention psy-PAD_Group_, as a part of the multimodal interdisciplinary setting of PsIAs. Hence, an expert workshop with clinicians from diabetology, psychosomatic medicine, and (psychological) psychotherapy, as well as patient representatives, will be conducted to discuss and adapt the psy-PAD_Group_ manual with a special focus on group psychotherapy aspects. The patient perspective assessed in work package 1 will directly inform the adaptation of the group intervention psy-PAD_Group_.

### Inclusion and Exclusion Criteria

#### Work Package 1: Patient Perspective

Patients aged between 18 and 69 years with a medically diagnosed type 1 or type 2 diabetes, an HbA_1c_ ≥7.5%, as well as pronounced diabetes-related distress (determined using the 5-item Problem Areas in Diabetes Questionnaire–short form with values≥11 [29] or clinical impression) will be included in the study to participate in the focus groups. Moreover, sufficient German language skills and cognitive abilities as well as written informed consent to participate in the study, are required. Exclusion criteria comprise a diagnosed type 3 diabetes or gestational diabetes, and severe comorbid diseases (eg, current oncological diseases that modify the indication for HbA_1c_). Patients will be excluded from the study in case of significant limitations for study participation, for example, dementia, severe depressive episode, psychosis, addiction, being bedridden, or the need for caregiving.

#### Work Package 2: Service Provider Perspective

Physicians or psychologists working in a diabetes clinic or in an outpatient practice specializing in diabetes will be included in the study to participate in the interviews (psychologists are generally present in diabetes clinics but not in outpatient practices). As it can also be assumed that, in outpatient practices, medical assistants will be involved in any referral processes (eg, handing out a screening form or the referral to established psychosomatic specialists, psychiatrists, or medical psychotherapists), they will also be interviewed about their needs regarding referral pathways. In a diabetes clinic, it is more likely that administrative staff will be involved in the referral pathways; hence, they will be included (independent of profession) to record their needs regarding the referral pathways to PsIAs.

#### Work Package 2: Expert Workshop

Persons will be included as experts if they belong to the physician or psychologist professions and have a special expertise in diabetology, psychotherapy, or group psychotherapy; have the status of an official patient representative; or experience diabetes themselves while being designated by patient self-help groups to represent the patient perspective.

### Sample Size

#### Work Package 1: Patient Perspective

The planned sample size of the focus groups results from empirically based recommendations for qualitative studies based on focus groups, which postulate a minimum sample size of 40 to achieve saturation [30]. Therefore, 4 focus groups of 10 (25%) patients each are planned.

#### Work Package 1: Service Provider Perspective

The planned sample size of the service provider interviews is set at 30. This is based on empirically founded recommendations postulating 22 to 24 as the minimum sample size to achieve saturation in individual interviews [31]. Due to the inclusion of various professions, we aimed to conduct 30 interviews: 16 physicians (53%), 4 psychologists (13%), 4 medical assistants (13%), and 6 administrative staff members (20%).

#### Work Package 2: Expert Workshop

For the expert workshop, the planned sample size is set at 8 clinically and scientifically active physicians or psychologists and 2 patients or patient representatives.

### Recruitment and Data Collection

#### Work Package 1: Patient Perspective

Patients with diabetes will be recruited from specialized diabetes clinics with a flyer containing information regarding the minDBe pilot study or by being actively asked to participate by the clinic staff.

#### Work Package 1: Service Provider Perspective

Service providers will be recruited from specialized diabetes clinics, outpatient practices specializing in diabetes, as well as quality circles for psychodiabetology.

#### Work Package 2: Expert Workshop

Clinically and scientifically active physicians and psychologists as well as patients or patient representatives will be recruited from the professional networks and quality circles for psychodiabetology as well as patient initiatives and associations for individuals with diabetes.

### Assessment and Data Analysis

#### Work Package 1: Patient Perspective

To assess the patient perspective, focus groups will be conducted based on a semistructured interview guideline. The interview guideline will cover the following topics: (1) current challenges in diabetes treatment, (2) needs and barriers regarding diabetes treatment, (3) referral pathways to PsIAs, and (4) needs regarding the new intervention psy-PAD_Group._ Each focus group will have a duration of 45 minutes and will be audio recorded.

#### Work Package 1: Service Provider Perspective

To assess the service provider perspective, interviews will also be conducted based on a semistructured interview guideline. For each service provider profession, a slightly edited interview guideline will be applied. The main topics comprise (1) psychological burden in patients with diabetes, (2) needs and barriers regarding psychological treatment for patients with diabetes, and (3) referral pathways to PsIAs. Each interview will have a duration of 20 to 25 minutes and will be audio recorded.

The audio recordings of both the patient focus groups and the service provider interviews will be transcribed by a professional commercial third party and analyzed using content analysis within the framework of interpretative text evaluations with MAXQDA. In this form of analysis, according to Kuckartz [32], the material is systematically described with regard to the individual categories, which are defined in connection with the research question and differentiated in the course of the analysis, with a particular focus on text comprehension and text interpretation. With the support of the MAXQDA analysis software, a deductive coding and summary of the first overarching main themes is carried out. During the development of the category system, the previously formed main themes are inductively expanded (from the data) by 2 raters, consensually agreed and supplemented by themes that appear to be particularly relevant when analyzing the text.

By recruiting from both cooperation networks and the routine care sector, we explicitly aim to include a broad range of perspectives of patients and service providers. The goal is to understand the typical issues arising in the referral process to PsIAs and how to best address them. Regarding the adaptation of the psy-PAD_Group_ intervention in work package 2, diverging perspectives will be discussed and solved by consensus.

#### Work Package 2: Expert Workshop

The expert workshop will be conducted as a moderated interactive discussion with a previously outlined set of topics and a duration of approximately 5 hours. The results of the expert workshop will be protocolled. Adaptations of the psy-PAD_Group_ manual as well as the required outline to implement the psy-PAD_Group_ manual in the modalities of PsIAs, will be based on the recommendations derived from the expert workshop.

During the data analysis and the development of the category system, previously formed main themes are inductively expanded from the data. For this purpose, 2 raters will agree consensually upon the categories and complementary themes that appear to be particularly relevant when analyzing the text. Once the category system has been created, the additional content is then classified by just one rater. The second rater can be consulted again if any ambiguities arise. To enhance dependability and confirmability, an audit trail will be maintained, including the key steps of data collection and transcription, coding decisions, and category development. All materials, including transcripts, coding frameworks, and analytic memos, will be securely stored to allow for transparency and traceability of the research process.

### Ethical Considerations

This study received ethics approval from the ethics committee of Justus-Liebig University Giessen—Faculty of Medicine (AZ 67/23) and is registered in the German Clinical Trial Register (DRKS00031873).

### Data Confidentiality

#### Work Package 1: Patient and Service Provider Perspective

The data collection of work package 1 comprises patient focus groups and interviews with service providers. The patient focus groups will be conducted in person, and the service provider interviews will be conducted in person or online. Online interviews will be carried out with the program Big Blue Button, which follows the security standards of the Justus-Liebig University. The focus groups and the interviews will be recorded with a suitable audio recording device with no internet connection. The audio recordings will be saved on an SD card or directly on the protected servers of the Justus-Liebig University. The recording device or SD card will be kept double locked and inaccessible to third parties on the premises of the Justus-Liebig University when not in use. The audio files will be transcribed by a professional commercial third party, which has extensive previous experience in conducting studies and research projects and has a comprehensive data protection concept for handling personal data. All references to persons or information that could be used to identify third parties are removed from the transcript during transcription. After these checks, the audio files are destroyed by both the third-party transcription service and the study center, so the data are available in pseudonymized form at this point. Further data processing and analysis of the pseudonymized transcripts is carried out using MAXQDA (VERBI GmbH) and SPSS (IBM Corp) for sample characteristics. In addition, personally identifiable data are collected in the form of contact details for planning the patient focus groups and service provider interviews, as well as the informed consent forms. The patient’s contact details will remain at the diabetes clinic at all times, and the list of focus group participants will be destroyed immediately after the focus group has been conducted. The contact details of the service providers as well as the informed consent forms, will be stored digitally on protected servers of the Justus-Liebig University or in paper form in the study center in a protected double-sealed manner for the duration of the study.

#### Work Package 2: Expert Workshop

The data collection of work package 2 comprises personal contact details, including the name and affiliation of the experts. As the participation in the expert workshop is to be made public, informed written consent is required in advance. The names and affiliations of the experts will be made public in the form of publications if informed written consent has been obtained. During the entire duration of the study, the data will be treated as personal data and stored exclusively in digitized form on protected servers and in paper form, double-sealed at the study center.

After completion of the study, all personally identifiable data will be deleted. The transcripts of the focus groups and interviews will be digitized for the required period of 10 years in accordance with data protection regulations and the “Guideline 17 Archiving—Guideline for Safeguarding Good Scientific Practice” [33] and stored on a hard drive specially for the purpose of archiving, double-sealed at the study center. The study will be conducted in accordance with the ethical requirements of the Declaration of Helsinki and in compliance with the standards of the ICH guideline for good clinical practice. Personal data of patients, service providers, and all other confidential information are subject to the provisions of the General Data Protection Regulation and the State and Federal Data Protection Act (Landesdatenschutzgesetz and Bundesdatenschutzgesetz). Participation in the study is voluntary at all times and only takes place after informed written consent.

## Results

### Work Package 1

Needs from the patient and service provider perspectives regarding referral pathways to PsIAs will be assessed. On the basis of these findings, referral pathways will be developed. Furthermore, in preparation for the upcoming minDBe main study, short questionnaires for patients and service providers will be developed to economically assess the acceptance and feasibility of the referral pathways as well as potential remaining access barriers. This will help determine whether the developed referral routes to PsIAs are successful in routine practice.

### Work Package 2

The intervention psy-PAD_Group_ will be adapted and manualized for a group setting to be integrated into the structures of PsIAs as part of an interdisciplinary multimodal treatment option.

Funding was acquired in July 2023. The data collection started in January 2025.

## Discussion

### Anticipated Findings

The results of the minDBe pilot study will comprise referral pathways to PsIAs using the perspective of patients and service providers, short questionnaires to economically assess these referral pathways, as well as a manual for the psy-PAD_Group_ intervention that can be implemented into the multimodal and interdisciplinary modalities of PsIAs. Given that PsIAs are a new care structure offering outpatient treatment, their role in the psychosomatic care landscape in Germany is yet not fully clear, and research in this regard is scarce. One key aspect of their success is how well PsIAs and their usefulness for both patients and service providers are perceived. By providing referral pathways, the minDBe pilot study is one of the first studies to address the practical challenges patients and service providers face when new care elements are introduced in an already complex health care system. More specifically, the needs of patients with diabetes and psychological burden and their treating physicians or diabetologists, are addressed when interdisciplinary and integrative treatment options are to be planned. Moreover, PsIAs require structured treatment options, which must be specifically designed to fit the modalities of PsIAs. For patients with diabetes and psychological burden, such a tailored treatment option does not exist yet. By adapting the psy-PAD intervention for a group setting, an important gap in the treatment of patients with diabetes and psychological burden is addressed. To date, patients with diabetes are primarily treated by their general practitioner or diabetologist, which tend to be more somatically focused, while psychological burden is treated by a psychotherapist. These treatments are usually not aligned with each other, even when they are received simultaneously. The psy-PAD_Group_ intervention aims to address this issue and presents a comprehensive treatment option integrating both somatic and psychological aspects of diabetes treatment.

The minDBe pilot study specifically aims at laying the foundation for the upcoming minDBe main study, by providing the necessary referral pathways, the short questionnaires, as well as the psy-PAD_Group_ manual. The minDBe main study aims to evaluate the referral pathways to PsIAs and prove their feasibility in clinical practice. Moreover, the main study will evaluate the psy-PAD_Group_ as part of an interdisciplinary psychotherapeutic treatment program in PsIAs by using a randomized controlled trial. The long-term goal of the minDBe overall study is the implementation of a diabetes-specific interdisciplinary multimodal psychotherapeutic treatment option into the new structures of PsIAs. To offer targeted care for the high-risk group of patients with diabetes, inadequate glycemic control, diabetes-related distress, and psychosocial burden, we intend to improve the overall treatment quality of patients with diabetes.

### Limitations

Regarding the proposed methodology of this study, several potential limitations should be acknowledged. For one, patients with gestational diabetes, other types of diabetes, or severe complications are excluded. Moreover, the diversity of the patient as well as the service provider sample is limited, which may restrict the generalizability of our findings. However, the goal of this qualitative pilot study is not statistical representativeness but theoretical saturation, which can also be achieved with less diverse samples when the research question is narrowly focused [31]. Mixed methods research inherently faces conceptual challenges, including the selection of appropriate methods for a given research question, the meaning of integrating different approaches, and the overall structuring of a mixed methods framework. In addition, qualitative research carries the risk of socially desirable responses, particularly when addressing sensitive topics. Given the time constraints for patient focus groups and service provider interviews, covering all aspects may not be possible.

## Abbreviations

HbA1c: hemoglobin A1c
PsIA: psychosomatic outpatient clinic
Psy-PAD_Group_: psy-PAD for a group setting

## Data Availability

The datasets generated or analyzed during this study are available from the corresponding author upon reasonable request.
